# Underestimated Craniomaxillofacial Fractures Due to Firework

**DOI:** 10.29252/wjps.10.3.46

**Published:** 2021-09

**Authors:** Mahdy Saboury, Noor Ahmad Latifi, Shahriar Saboury, Sona Akbarikia, Fatemeh Latifi, Mohsen Khaleghian, Mohammad Hosein Kalantar Motamedi

**Affiliations:** 1 Department of Plastic and Reconstructive Surgery, School of Medicine, Iran University of Medical Sciences, Tehran, Iran; 2 Department of General Surgery, School of Medicine, Iran University of Medical Sciences, Tehran, Iran; 3 Department of Radiology, School of Medicine, Tehran University of Medical Sciences, Tehran, Iran; 4 Department of Oral and Maxillofacial Surgery, School of Dentistry, Shahid Beheshti University of Medical Sciences, Tehran, Iran; 5 Department of General Surgery, School of Medicine, Iran University of Medical Sciences, Tehran, Iran; 6 Department of Craniomaxillofacial Tra-uma Research, School of Medicine, Baqiyatallah University of Medical Sciences, Tehran, Iran

**Keywords:** Trauma, Maxillofacial, Fracture, Firework, Explosive agents

## Abstract

**BACKGROUND:**

Iranian people celebrate the last Wednesday of the year also known as Chahar Shambeh Soori (CSS) using low explosive pyrotechnics classified as fireworks. Mishaps and accidents are common and maxillofacial fractures may occur which have a negative impact on the quality of life. This study aimed to assess maxillofacial fractures (fx) caused by explosive agents.

**METHODS:**

This cross-sectional descriptive study assessed 283 patients suffering maxillofacial fxs caused by explosive agents during CSS ceremonies between 2009 and 2019 referred to our craniomaxillofacial (CMF) surgery center. The data assessed included age, sex, cause, type, site, and severity of injury, fracture patterns, treatment modalities, and complications. All maxillofacial injuries were evaluated and treated by Craniomaxillofacial staff surgeons.

**RESULTS:**

Among 283 patients, 72.8% (206) and 27.2% (77) were men and women, respectively. The mean age of patients was 17.35 years. The most common maxillofacial fracture was in the mid-face; with the distribution of fractures being: 39.9% zygomatic fractures, 32.1% nasal bone fractures, 63.2% dentoalveolar fracture, 43.1% Le Fort (Le Fort I, Le Fort II, Le Fort III), 31.4% orbital, and 43.1% mandible fractures. The most frequent type of treatment was Open Reduction and Internal Fixation (ORIF) (77.4%).

**CONCLUSION:**

The most common site of maxillofacial fractures and most frequent treatment used were similar to military or ballistic injuries. ORIF was common treatment.

## INTRODUCTION

Fireworks a types of low explosive pyrotechnic devices are used in various ceremonies such as New Year’s celebrations, the Fourth of July, Halloween, etc.^1^^-^^3^. The Persians celebrate CSS using low explosive pyrotechnics which dates back to 1725 BC, held on the last Tuesday night of the year (based on the Persian calendar). People celebrate by lighting fires and jumping over (as a gesture to ward off evil and disaster and also to fulfill their prayers)^1^^-^^2^. Unfortunately, in recent years, teenagers cast dangerous incendiary (explosive materials such as picnic gas capsules) into the bonfire. Youngsters and children make and use homemade explosive materials (such as fire crackers, bottle rockets, 180s, grenades, etc.) sometimes with faulty detonation. They then pay staggering sums of money every year for treatment of injuries such as facial burns, injuries, amputations, and physical disabilities^1^. 

 Facial burns are among the most painful CSS injuries, resulting in scars; and have a dire impact on the quality of life. In 2004, 11 million people were traumatized by burns globally^2^, and it is a major problem in most parts of the world. About 1.4 to 2 million burns occur annually in the United States, and 70,000 patients are hospitalized. Facial trauma causes irreparable damage to the patient, which can lead to long-term physical and mental problems^3^. Burn traumas are also one of the most important causes of mortality (5% or more of the total number of hospital patients)^4^. In general, the craniomaxillofacial (CMF) trauma occurs following a wide variety of traumas. The most common causes of CMF trauma include vehicle accidents and explosive agents^5^^-^^9^.

Head and neck fractures include orbital, zygomatic, Le Fort, mandibular, condylar, and alveolar fractures.

One important injury caused by the explosion of incendiary materials is orbital fracture. Anteriorly, the orbital rims consist of a thick bone. The middle third of the orbit consists of a thin bone, and the bone structure thickens again in the posterior portion of the orbit. The orbital bone structure is thus analogous to a shock-absorbing device where the middle portion of the orbit breaks first, followed by the rim, both absorbing energy and protecting the posterior third from displacement as well as protection `of the globe from rupturing^8^.

Many patients with CMF trauma also experience trigeminal and infra-alveolar nerve injuries. These injuries are mainly due to the displacement of the fracture segments. There are many studies reporting a relationship between Maxillofacial (MF) fractures and nerve damages such as orbital fractures and the superior orbital fissure syndrome or the orbital apex syndrome, zygomatic fractures and infra-orbital nerve damage or mandibular fractures with inferior alveolar nerve damage^6^^-^^12^. The prevalence of inferior alveolar nerve paresthesia following mandibular fracture has been reported to be 18% to 91%. Permanent inferior alveolar nerve paresthesia has also been reported to be 2%–47%. In general, the costs of the disease are classified into two categories: direct costs and indirect costs. Direct costs are directly spent on providing health care to the patient, which include direct medical and non-medical costs. Indirect costs of a disease, on the other hand, are the costs associated with the patient’s lost production due to illness. This complication is considered an important health and medical problem, especially in developing countries, facing constraints of highly qualified specialists and specialized medical equipment^13^^-^^15^.

Due to the importance of high treatment and maintenance costs such damages inflict on families, the health care system and the community, we have evaluated the damage caused by incendiary substances and fireworks. Meanwhile, this is the first study that evaluated the pattern of CMF fractures caused by New Year’s Persian Fire Festival.

## METHODS

This cross-sectional retrospective study was performed to assess CSS ceremony-related MF fractures in patients between 2009 and 2019 referred to our plastic and craniomaxillofacial surgery center. 

This study was confirmed by Ethical Committee of Iran University of Medical Sciences and Baqiyatallah University of Medical Sciences: IR.BMSU.BAQ.REC.1398.046. 

The records of all patients injured during the mentioned period were extracted. The extracted data were assessed and only CMF fractures patients were included. All craniomaxillofacial injuries were evaluated and treated by craniomaxillofacial surgeons. All eligible samples were included in the study, and sampling was performed by census sampling and a researcher-made questionnaire.

Gender, age, site of injury, severity of injury, fracture patterns, treatment modalities, and complications were analyzed. A checklist was used for data collection and assessed after completion. All data were analyzed using SPSS 20 software (Chicago, IL, USA). A *P*-value ≤ 0.05 was considered as statistically significant.

## RESULTS

*Demographics*

During the 10-year period, 283 patients with CMF fractures due to CSS injuries were admitted to our center. Table 1 reports the number of cases per year. The highest number of cases was related to 2011 with 34 cases followed by 2009 with 33 cases.

The demographic results indicated that 72.8% (206) of patients were men.

The mean age was 17.35 yr, ranging from 9 to 26 years. The average age of men was 17.78 yr and that of women was 16.21 years (Table 2).

Moreover, 50.5% of patients were between 15 and 20 years. Note that only 1.4% of patients were under the age of 10 years. Thus, 97.2% of patients were between 10 and 25 yr old.

*Distribution of Fractures*

Upper face injuries included fractures of the orbital rim, orbital roof, and frontal sinus. Midface fractures were defined as superior zygomaticofrontal suture and the area from the superior orbital rim to the maxillary occlusal plane. The lower face injuries were related to mandibular fractures.

Table 3 lists the distribution of MF fractures. The most common MF fxs were mid-face fractures followed by lower face fractures.

There was a statistically significant relationship between gender and type of fracture, in the upper and midfacial zones (Table 4).

**Subcondylar** fracture was seen in 50 patients and the frequency of symphysis fracture was 7.4%. All pan-facial fracture cases were female (Table 5).

*Associated injuries*

Diplopia and visual acuity changes were seen in 20.5% and 18.7% of patients, respectively. Table 6 presents the acute complications seen in MF fractures patients. 

*Treatment*

The most common type of treatment (77.4%) was Open Reduction and Internal Fixation (ORIF), followed by InterMaxillary Fixation (IMF) (75.6%). Obviously, one patient may have received more than one treatment modality. The frequency of total therapeutic interventions is shown in Table 7.

According to the type of the MF fractures, specific treatments are listed in Tables 8-11.

*Late complications*

This study showed that the most common type of complication in MF fractures related to fireworks was malocclusion followed by osteomyelitis, 11.7% and 6.7%, respectively. Nonunion and malunion were not significant. Also, 2% of our patients had infra-orbital nerve injury and 1.4% experienced infra-alveolar paresthesia. There was a statistically significant relationship between the type of MF fractures and aforementioned complications (Table 12).

In our study, the mortality rate was 2.1% all being women (*P*=0.001).

**Table 1:** The number of cases per year

**Year**	**2009**	**2010**	**2011**	**2012**	**2013**	**2014**	**2015**	**2016**	**2017**	**2018**	**2019**	**Total**
**Number**	33	29	34	25	22	28	29	16	25	22	20	283
**%**	11.7	10.2	12	8.8	7.8	9.9	10.2	5.7	8.8	7.8	7.1	100

The demographic results indicated that 72.8% (206) of patients were men.

**Table 2:** Age description (classified) of patients

**Age(yr)**	< 10	10-15	15-20	20-25	> 25	Total
**Number**	4	79	143	53	4	283
**%**	**1.4**	**27.9**	**50.5**	**18.7**	**1.4**	**100**

**Table 3:** Fractures site distribution

**Type**	Upper face	Mid face	Lower face
**Number (%)**	**99 (35 %)**	**227 (80.2 %)**	**122 (43.1 %)**

**Table 4:** Frequency of upper and mid facial fractures

SITE of fractures	SEGMENTS	M	F	%	*Pearson Chi-Sq. Test ** Fisher Exact Test
Frontal bone	Anterior Table	18	4	7.8	* *P* =0.001 ** *P* =0.001
	Posterior Table	0	4	1.4	
	Anterior and Posterior Tables	3	8	3.9	
Orbit	Medial Wall	5	0	1.8	* *P *=0.001 ** *P* =0.001
	Floor	54	4	20.5	
	Medial Wall and Floor	7	19	9.2	
Nasal bone		66	25	32.2	* P =0.944 ** *P* =0.971
Ethmoidal bone		18	12	10.6	* P =0.002 ** *P* =0.005
Zygoma	Arch	19	4	8.1	* *P* =0.001 ** *P *=0.001
	Body	77	13	31.8	
Dentoalveolar	Upper	104	27	46.3	* *P* =0.068 ** *P* =0.066
	Lower	32	26	17	
Le Fort	Le Fort I	60	15	26.5	* *P* =0.041 ** *P* =0.045
	Le Fort II	32	9	14.5	
	Le Fort III	2	4	2.1	

**Table 5:** Distribution of Mandibular and Pan facial fractures

SITE of fractures	**segments**	M	F	%	*Pearson Chi-Sq. Test ** Fisher Exact Test
Mandible	Angle	14	5	6.7	* *P* =0.12 ** *P* =0.151
	Condyle	10	6	5.7	
	Subcondyle	36	14	17.7	
	Coronoid	3	0	1.1	
	Body	3	6	3.2	
	Para symphysis	4	0	1.4	
	symphysis	10	11	7.4	
Pan facial		0	12	4.2	* *P* =0.001 ** *P* =0.001

**Table 6:** Acute complication related to MF fractures

Type		Gender		Total	*P*-value
		Male	Female		
Dural laceration	Number	4	12	16	^*^ *P*= 0.001
					^**^ *P* = 0.001
	%	1.4	4.2	5.6	
Rhinorrhea	Number	4	8	12	^*^ *P*= 0.002
	%	1.4	2.8	4.2	^**^ *P* = 0.004
Otorrhea	Number	3	8	11	^*^ *P*= 0.002
	%	1.1	2.8	3.9	^**^ *P* = 0.003
Diplopia	Number	45	13	58	^*^ *P*= 0.357
	%	15.9	4.6	20.5	^**^ *P* = 0.411
Lacrimal Duct Injury	Number	12	12	24	^*^ *P*= 0.009
	%	4.2	4.2	8.4	^**^ *P* = 0.015
Enophthalmos	Number	28	20	48	^*^ *P*= 0.014
	%	9.9	7.1	17	^**^ *P* = 0.020
Visual Acuity Change	Number	35	18	53	^*^ *P*= 0.220
	%	12.4	6.4	18.7	^**^ *P* = 0.233
Malocclusion	Number	21	12	33	^*^ *P*= 0.209
	%	7.4	4.2	11.7	^**^ *P *= 0.216

* Pearson Chi-Sq. Test ** Fisher Exact Test

**Table 7:** Treatment distribution

Type	ORIF	Canthal reattachment	Trans nasal canthopexy	Close reduction	Observation	IMF
Number	219	30	30	43	35	214
percentage	77.40%	10.60%	10.60%	15.20%	12.40%	75.60%

**Table 8:** Distribution of treatment for frontal bone fracture

**Percentage**	**Number**	**Type of treatment**	**Area**	**Fracture**
**36.4**	8	Reconstruction with Pelvic bone	Anterior Table	Frontal bone
**40.9**	9	Observation		
**59.1**	13	ORIF		
**100**	4	Reconstruction with Pelvic bone	Posterior Table	
**100**	4	ORIF		
**100**	11	Reconstruction with Pelvic bone	Anterior and Posterior Tables	
**100**	11	ORIF		

**Table 9:** Distribution of treatment for Orbital fractures

**Percentage**	**Number**	**Type of treatment **	**Area**	**Fracture**
**20**	1	Reconstruction with Pelvic bone	Medial wall fractures	Orbital fractures
**20**	1	ORIF		
**22.4**	13	Reconstruction with Titanium Mesh	orbital floor fractures	
**53.4**	31	Reconstruction with Pelvic bone		
**100**	58	ORIF		
**69.2**	18	Reconstruction with Pelvic bone	Medial wall and floor	
**96.2**	25	ORIF		

**Table 10:** Distribution of treatments for Le Fort fractures

**Percentage**	**Number**	**Type of treatment **	**Type**	**Fracture**
**9.3**	7	Reconstruction with Pelvic bone	Le Fort I	Le Fort
**10.7**	8	Reconstruction with Titanium Mesh		
**100**	75	ORIF		
**7.3**	3	Reconstruction with Titanium Mesh	Le Fort II	
**19.5**	8	Reconstruction with Pelvic bone		
**90.2**	37	ORIF		
**66.7**	4	Reconstruction with Pelvic bone	Le Fort III	
**100**	6	ORIF		

**Table 11:** Distribution of treatments for mandibular fractures

**Percentage**	**Number**	**Type of treatment **	**Area**	**Fracture**
**100**	19	IMF	Angle	Mandible
**100**	19	ORIF		
**100**	16	IMF	Condyle	
**100**	16	ORIF		
**16**	8	Reconstruction with Pelvic bone	Subcondyle	
**84**	42	IMF		
**94**	47	ORIF		
**10**	5	Close reduction		
**100**	3	IMF	Coronoid	
**100**	3	Close reduction		
**100**	4	Close reduction	Para symphysis	
**19**	4	IMF	Symphysis	
**28.6**	6	Close reduction		
**19**	4	ORIF		
**71.4**	15	Observation		
**66.7**	6	IMF	Body	
**33.3**	3	Close reduction		
**66.7**	6	ORIF		

**Table 12:** The correlation between type of fracture and complications

**Type of fracture**	**Frontal**		**Le fort**		**Pan facial**		**Mandible**		**Mandible**	
**Type of complication **	Osteomyelitis		Malocclusion	Osteomyelitis	Malocclusion	Osteomyelitis	Osteomyelitis		Malocclusion	
***P*** **-value**	^*^ *P*= 0.001	^**^ *P *= 0.001	^*^ *P*= 0.001	^**^ *P* = 0.001	^*^ *P*= 0.001	^**^ *P* = 0.001	^*^ *P*= 0.007	^**^ *P* = 0.008	^* ^ *P*= 0.004	^**^ *P* = 0.001

* Pearson Chi-Sq. Test ** Fisher Exact Test

## DISCUSSION

This study assessed the MF injuries caused by explosive agents used in CCS ceremonies. The highest number of cases was related to 2011 with 34 cases followed by 2009 with 33 cases. The demographic results revealed that 72.8% (206) of patients were men and 27.2% (77) were women. The mean age of patients was 17.35 yr with the age range being 9 to 26 years. The average age of men was 17.78 yr and for women was 16.21 years. Moreover, 50.5% of patients were in an age range of 15-20 years. Only 1.4% of patients were under 10 yr old. Thus, 97.2% of patients were between 10 and 25 yr old. Aghaee et al evaluated the epidemiology of firework injuries in the CSS ceremonies, and reported 83.2% of patients were male and 16.8% female with an average age of 20.9 ±11.12 years. The age group of 15-24 yr claimed the highest number of patients ^16^ that was similar to our study. 

The sites of the body injured in CSS ceremony have been evaluated in the related literature. The highest percentage of patients were in the age group of 16-20 yr with the main burn victims being men (81%). The highest rate of destruction occurred in the hands, head, and face (46%). They reported one dead^17^. Puri et al. reported the hands to be the main site of injury in 80% of the cases they studied, as with other studies in Australia, Saudi Arabia, England, India, Ireland, and Denmark^3^. The results proposed the logical reason that people between the ages 21-30 yr are further participated in fireworks and more exposed with low explosive pyrotechnic devices, subsequently leading to higher rates of trauma specially MF fractures.

Moreover, the results of other studies such as predominance of men and age group are in line with ours. Nevertheless, because of our study field, sites of injury were different. The most common sites of injury were the hand and foot, followed by the eye and face with a lower incidence. 

To the best of our knowledge, this is the first study focusing on MF fxs sustained in CSS ceremonies. In this study, the most common MF fxs were mid-face fxs followed by lower face fxs and then upper face fxs. 

There were 39.9% zygomatic fractures, 32.1% nasal bone fractures, 63.2% dentoalveolar fracture, 43.1 % Le Fort region (Le Fort I, Le Fort II, Le Fort III), 31.4% orbital fxs, and 43.1% mandible fxs. Surprisingly, our study findings were similar to ballistic studies.^9^^,^^18^

 In other studies Nasal fractures were the commonest site of fractures, but our results showed that dentoalveolar was the most common. This difference may be due to this fact that the cause of injury in our patients was low explosive pyrotechnic devices, in contrast to military studies with more powerful materials.

In our study, the most common fracture sites in the mandible were the subcondyle fracture followed by the symphysis and angle region. The incidence of mandibular fracture varied in other studies^10^^,^^11^, but similar findings were seen in some other studies ^18^.

The management of MF fractures correlate with novelties, knowledge, and materials. In this study ORIF (77.4%) was the most common treatment. Typically, surgeons select the open reduction and plate osteosynthesis technique as a replacement for closed reduction, as this procedure has numerous benefits including rapid return of function, early recovery, patient comfort, and segment stability^19^^-^^21^. 

This study showed that the most common type of complication in MF fractures related to fireworks was malocclusion followed by osteomyelitis, 11.7% and 6.7%, respectively. They had a statistically significant relationship with frontal, Le fort, mandible, and pan-facial fractures (*P*<0.05). Other studies found similar results^22^^-^^23^. Malunion and infections were measured as the most common complications and major cause of morbidity^22^ and Ophthalmic injuries were presented in about 20% of midfield traumas, also osteomyelitis was the most prevalent post-operative complication^24^.

## CONCLUSION

The pattern of maxillofacial injuries and the therapeutic interventions used for their management were similar to ballistic or military maxillofacial injuries that most commonly caused mid-face fractures. Moreover, ORIF was the most common therapeutic method. Assessment of the real burden of injuries and their impact on healthcare system, can aid the pursuit of preventive measures and their mandatory use on CSS.

## CONFLICT OF INTEREST

The authors declare that there is no conflict of interests.
